# Discovering Hidden Connections among Diseases, Genes and Drugs Based on Microarray Expression Profiles with Negative-Term Filtering

**DOI:** 10.1371/journal.pone.0098826

**Published:** 2014-06-10

**Authors:** Jain-Shing Wu, E-Fong Kao, Chung-Nan Lee

**Affiliations:** 1 Department of Computer Science and Engineering, National Sun Yat-Sen University, Kaohsiung, Taiwan; 2 Department of Medical Imaging and Radiological Sciences, Kaohsiung Medical University, Kaohsiung, Taiwan; University of Jaén, Spain

## Abstract

Microarrays based on gene expression profiles (GEPs) can be tailored specifically for a variety of topics to provide a precise and efficient means with which to discover hidden information. This study proposes a novel means of employing existing GEPs to reveal hidden relationships among diseases, genes, and drugs within a rich biomedical database, PubMed. Unlike the co-occurrence method, which considers only the appearance of keywords, the proposed method also takes into account negative relationships and non-relationships among keywords, the importance of which has been demonstrated in previous studies. Three scenarios were conducted to verify the efficacy of the proposed method. In Scenario 1, disease and drug GEPs (disease: lymphoma cancer, lymph node cancer, and drug: cyclophosphamide) were used to obtain lists of disease- and drug-related genes. Fifteen hidden connections were identified between the diseases and the drug. In Scenario 2, we adopted different diseases and drug GEPs (disease: AML-ALL dataset and drug: Gefitinib) to obtain lists of important diseases and drug-related genes. In this case, ten hidden connections were identified. In Scenario 3, we obtained a list of disease-related genes from the disease-related GEP (liver cancer) and the drug (Capecitabine) on the PharmGKB website, resulting in twenty-two hidden connections. Experimental results demonstrate the efficacy of the proposed method in uncovering hidden connections among diseases, genes, and drugs. Following implementation of the weight function in the proposed method, a large number of the documents obtained in each of the scenarios were judged to be related: 834 of 4028 documents, 789 of 1216 documents, and 1928 of 3791 documents in Scenarios 1, 2, and 3, respectively. The negative-term filtering scheme also uncovered a large number of negative relationships as well as non-relationships among these connections: 97 of 834, 38 of 789, and 202 of 1928 in Scenarios 1, 2, and 3, respectively.

## Background

In recent years, a considerable volume of biomedical literature has been published, covering a range of research topics including the functional genome, epigenetic modifications, and mRNA and protein expression profiles. This research outlines the functions, mechanisms, and behaviors of genes in regular cellular processes. A considerable quantity of useful data has been obtained; however, still more information awaits discovery. For example, one study may identify genes related to colon cancer from a microarray expression profile while other researchers observe changes in the gene expression value through a study on drug response. Thus, the same genes may possess hidden connections based on the specifics of the disease and drugs in question. Swanson [1] referred to this situation as undiscovered public knowledge (UPK) residing in two previously published collections of complementary literature. UPK can provide useful new information of scientific interest. Swanson [1], [3] combined information related to target terms (A), intermediate concepts (B), and other terms (C) from two discrete sources, thereby providing new directions from which to obtain hidden relationships (A->B, B->C  = > A->C).

A variety of methods have been developed for the extraction of UPK from previously published studies. Spasser [2] applied the concepts proposed by Swanson in other areas, such as library informatics. Lamb [4] proposed a systematic method for the construction of connectivity maps comprising a reference collection of gene expression data from human cells treated with bioactive small molecules. However, the gene-expression signature (one file of up-regulated genes and one file of down-regulated genes derived from a transcriptional profiling experiment) must be prepared in advance.

Butte and Kohane [5] used the unified medical language systems (UMLS) meta thesaurus, Gene Expression Omnibus (GEO) files, and publically available gene expression profiles to construct connectivity maps which link biologically significant terms (phenotypes, diseases, environmental and experimental conditions) with related genes. They also performed a t-test to determine the relationship between datasets and concepts, the values of which were used to identify genes of significance from within the gene expression profiles. However, the integration of data can be difficult when experiments are performed in different laboratories, processing different samples, on different experimental platforms [6]. For example, the results obtained using cDNA microarrays cannot be integrated using GEO files. Furthermore, it is difficult to judge relationships when two conceptually similar experiments provide opposing relationships via the expression profiles.

A set of interactive software and database search strategies called ARROWSMITH [7] has been developed to reveal plausible hypotheses-linking findings from across a range of specialties. This system collects studies from MEDLINE, the titles of which contain term A, which are stored as File A. In the same manner, studies are collected with titles containing term C, which are stored as File C. A list is then compiled from Files A and C using common term B (words or phrases). A 5000 item stop-list is then used to filter out uninteresting terms from B before scrutinizing list B for plausible links between A and C. For each B term, the system generates AB files with titles containing A and B terms as well as BC files with titles containing B and C terms. This tool focuses on titles containing A or C terms, which are inherently limited with regard to the number of words employed. In addition, the system only finds co-occurrences of A and B or B and C in the title. Finally, to generate AB or BC files, ARROWSMITH employs manual editing in its search for plausible links between A and C via B terms. This is a novel approach to discover relationships between clinical conditions and physiological states through the analysis of MEDLINE document titles.

Homayouni et al. [8] proposed the use of latent semantic indexing (LSI), a vector space model capable of automatically identifying relationships among genes from titles and abstracts listed as MEDLINE citations. A semantic gene organizer (SGO) first retrieves LocusLink PMID abstracts from which it extracts gene-related documents. The SGO then creates terms using a gene-document matrix followed by singular value decomposition (SVD) for the creation of a low-rank approximation matrix. A vector space model is then used by the SGO to identify relationships among genes or between genes and keywords. However, when a large number of abstracts have been retrieved, the gene-document matrix can become unwieldy. Determining appropriate restrictions to control the size of the dictionary has proven difficult.

Another common approach to identify the relationships among biomedical concepts is the co-occurrence method, based on the theory that if two biomedical concepts occur in the same study, they might be related.

Frijters et al. [9] proposed a co-occurrence method called CoPub, which uses multiple genes of humans and mice to generate lists of keywords from several biomedical databases. These lists link to MEDLINE abstracts, in which the keywords are highlighted. In 2010, Frijters et al. [10] applied CoPub to produce connectivity maps by employing A to B and B to C relationships to find hidden relationships A to C. However, unlike ARROWSMITH, A, B and C represent biological terms such as diseases, drugs, and biological processes. Using this approach, a collection of abstracts related to the biological terms A and B is first obtained, and then a second collection from B and C is also generated. The relationship between the two collections is then calculated and a connectivity map is then produced to display the results. This is a powerful method of revealing hidden connections. CoPub uses catalog terms instead of original terms to retrieve related studies; however, this approach occasionally results in high recall but low precision. For example, terms such as “acute lymphoblastic leukemia” and “acute myeloid leukemia” are classified using simplified terms, such as “leukemia”, despite the fact that studies related to “chronic lymphoblastic leukemia” and “chronic myeloid leukemia” are also retrieved. When focusing on genes related to AML or ALL, studies related to CML and CLL are regarded as false positives and must be filtered out. In addition, terms such as “colorectal cancer” are often classified within a specific catalog such as “colon cancer”. CoPub deals only with the term “colon cancer” but not the term “colorectal cancer”, which can often appear in the same studies. As a result, many studies that could be highly relevant to the input terms (disease names) are often missed. Furthermore, CoPub deals only with co-occurrences, without any consideration of the status of the relationship (whether it is a positive relationship, negative relationship, or non-relationship).

Li et al. [6] proposed a novel algorithm to build disease-specific drug-protein connectivity maps using molecular interaction networks and PubMed [11]. Proteins were imported as “seeds” for use in the expansion of a protein network list. The list of proteins is used to generate queries for the identification of relevant abstracts as well as a “drug filtering criteria” with which to identify drugs of interest. After combining the disease-related proteins and drug list, cluster analysis is used to generate a disease-specific connectivity map. Unfortunately, this method requires manual intervention for the selection of proteins to seed the list as well as specific drug filter criteria.

The above methods were developed to facilitate the discovery of UPK; however, additional information related to microarray data remains largely unexplored. Moreover, most of these methods are unable to adequately deal with negative relationships or non-relationships among the entities in question. If a relationship between a particular gene and a disease or drug is reported in an abstract, but that relationship is in fact negative, the abstract must be removed from the collection. For example, “hSP (human spasmolytic polypeptide), the domain-duplicated homolog of pS2 protein is co-expressed with pS2 in stomach but not in breast carcinoma” details a negative relationship between “hSP” and “breast carcinoma”. Many studies [12]–[14] have pointed out the importance of effectively dealing with studies that exhibit negative relationships or non-relationships.

This study employed microarray expression profiles for the identification of intermediate terms (genes). In the spotted microarray technique, the genes of interest must be selected first and corresponding probes are then printed to the glass for the experiment [15]. For in situ synthesized microarrays, such as Affymetrix GeneChips [16], microarray chips are produced for the observation of several topics. In this case, probes are designed according to pre-selected genes, thereby considerably narrowing the scope. Microarray expression profiles also provide evidence of the relationship between genes and various topics of interest. The main objective of this paper was to use microarray profiles for the discovery of relationships among diseases, genes, and drugs, whereupon biomedical literature can be used to confirm the obtained results. The proposed system is not intended to be a replacement for existing approaches such as CoPub, but to be a complementary approach to obtaining hidden connections as well as an effective means of dealing with documents that exhibit negative relationships or non-relationships.

## Results

Three scenarios were used to verify the performance of the proposed method. Scenario 1: Disease- and drug-related gene lists are obtained from disease- and drug-related microarray expression profiles in which the names of diseases and drugs are also adopted. Scenario 2: Various disease- and drug-related microarray expression profiles are used to obtain disease- and drug-related gene lists. Scenario 3: A disease-related gene expression profile is adopted and drug-related gene files are obtained from the PharmGKB website [17] (http:/www.pharmgkb.org/index.jsp).

In the event that GEPs are used, then a gene selection method called the disease oriented gene selection algorithm (DOFA) [18] is first used to obtain disease- or drug-related gene lists. DOFA analyses each gene in the GEP. If all expression values of samples at one gene are close to the mean value of their classes and far from those of other classes, then this gene is regarded as important. If the expression values of samples at one gene make it difficult to distinguish the classes, then this gene is viewed as unimportant. In this manner, DOFA filters out unimportant genes and then uses a genetic algorithm (GA) for the selection of genes capable of precise classification of the samples. These genes are deemed to be related to the disease or drugs in question. Further details of DOFA are provided in Algorithm S1. After obtaining gene lists related to the diseases or drugs in question, synonyms of the gene names in the lists are obtained from the Genebank. These names and the input terms (disease or drug names) are then used to fetch abstracts and titles from PubMed, which are evaluated according to relevance using a weight function. The abstracts in PubMed are fetched using a search engine based on the co-occurrence method; therefore, they do not always display positive relationships. Thus, the proposed method examines all retrieved abstracts and removes those that are unrelated or evidence a negative relationship. Finally, the remaining abstracts are used to identify relationships indicative of hidden connections among genes, diseases, and drugs. Further details of the proposed method are outlined in the Methods section, and details of the experiments are presented in the following. And all related softwares, data, analysis script and user's manual can be found in [40].

### Scenario 1

DOFA was used for the selection of dominant genes from the disease- and drug-related GEPs. This scenario included “lymphoma cancer” and “lymph node cancer” as the target disease terms and “cyclophosphamide” and “cytophosphane” as the target drug terms. The weight function was applied to the disease and drug terms to evaluate the relevance of the retrieved abstracts. The lymphoma cancer dataset reported by Shipp et al. [19] was selected as the disease-related GEP. This dataset contains 77 samples, each of which contains 7129 genes. We also adopted the drug-related GEP in [19], containing 58 samples, each of which contains 7129 genes.

**Figure 1:**
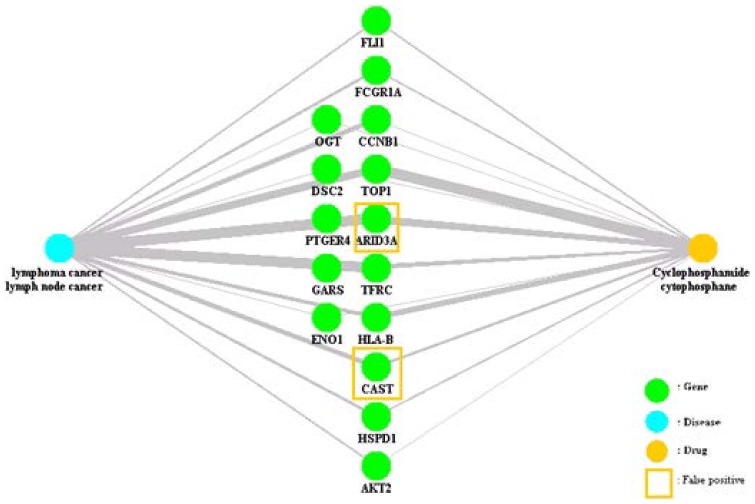
Connectivity map of the disease “lymphoma cancer, lymph node cancer” and the drug “cyclophosphamide, cytophosphane” obtained using the proposed method. The node marked in blue represents the disease node; the nodes marked in green represent the gene nodes; the node marked in yellow represents the drug node. The thicker the edge is connecting diseases and genes or drugs and genes, the stronger the relationship. The genes that are obtained using the proposed method are connected to both diseases and drugs. The genes bounded in a yellow box are the false-positive genes.

DOFA identified 54 genes from the disease-related GEP and 33 genes from the drug-related GEP, which were used to retrieve related abstracts from the PubMed website. DOFA identified 15 genes with hidden connections between the input disease terms “lymphoma cancer, lymph node cancer” and the input drug terms “cyclophosphamide, cytophosphane”, as shown in Fig. 1. The node marked in blue represents the disease node; the node marked in green represents the gene nodes; and the node marked in yellow represents the drug node.

The gene “TOP1” is an intermediate node between the disease and drug of interest. Kancherla et al. [20] claimed that “the authors report Phase I data of topotecan and etoposide combination for patients with recurrent or refractory non-Hodgkin lymphoma and correlation of topoisomerase-DNA complex formation to clinical response”. In addition, Minagawa et al. stated, “In 8 cases whose samples could be obtained before and after cyclophosphamide (CAP), topoisomerase I (topo I) activity significantly increased after CAP therapy” [21]. These statements attest to the relationship between the diseases and genes as well as between the drugs and genes.

In contrast, the gene “CAST” was a false positive because the term “cast” is a commonly used verb. The gene “ARID3A” was also a false positive because its alias “bright” is a commonly used adjective.

**Figure 2:**
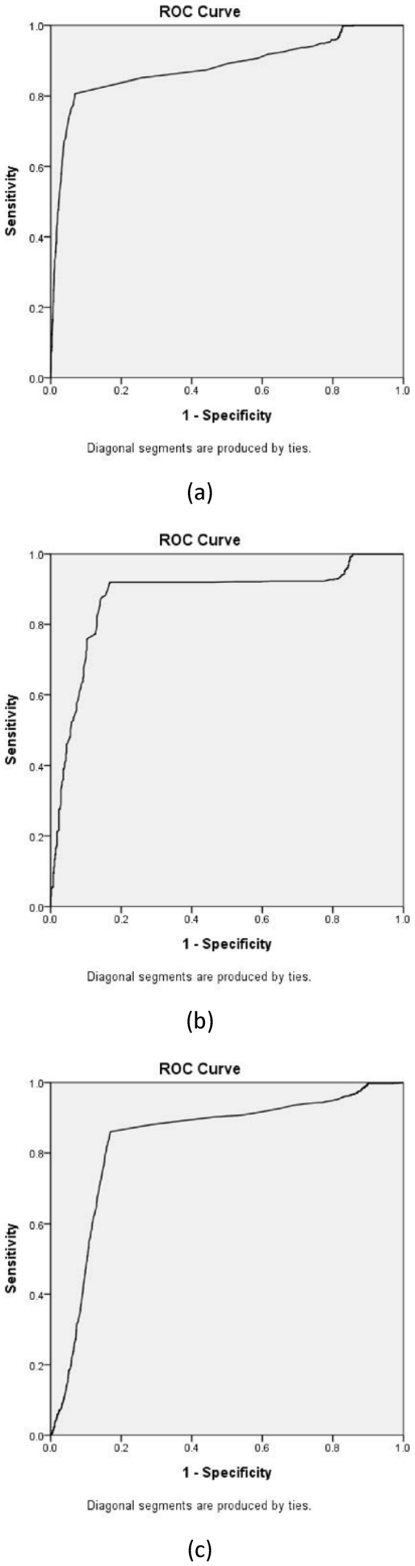
ROC results of Scenario 1, Scenario 2, and Scenario 3. The variable “score” is the related score of the study calculated using the proposed method with the weight function and the negative-term filtering scheme with zero as the threshold value. Figure 2(a) shows the ROC results of Scenario 1. The sensitivity and the specificity of Scenario 1 results are 81.98% and 92.21%, respectively. The area under curve (AUC) in this case is 0.883, and the 95% confidence interval (CI) is between 0.873 and 0.893. (b) The sensitivity and the specificity of Scenario 2 results are 92.54% and 83.27%, respectively. AUC in this case is 0.882, and the 95% CI is between 0.862 and 0.899. (c) The sensitivity and the specificity of Scenario 3 results are 83.78% and 83.82%, respectively. AUC in this case is 0.822, and the 95% CI is between 0.809 and 0.834.

A receiver operating characteristic (ROC) curve was used to evaluate the ability of the proposed method to uncover hidden connections among previously published studies. All retrieved abstracts are manually checked for relationships between diseases and genes or drug and genes to provide a gold standard with which to evaluate the results obtained after using the weight function and negative-term filtering scheme. Finally, an ROC curve is drawn with zero as the threshold value. To draw the ROC curves, this study used the statistical software, Statistical Product and Service Solutions (SPSS), which is commonly used for statistical calculation. Figure 2 (a) presents the ROC results of Scenario 1, the sensitivity and specificity of which were 80.7% and 93.10%, respectively. The area under the curve (AUC) in this case was 0.881 with a 95% confidence interval (CI) of between 0.862 and 0.9. Data of three scenarios for drawing three ROC curves are provided in Data S1 and also available in [40].

**Figure 3:**
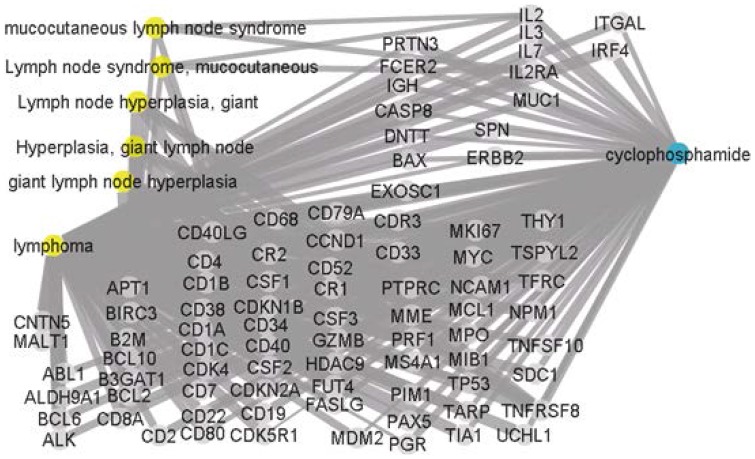
Connectivity map of the disease “lymphoma cancer, lymph node cancer” and the drug “cyclophosphamide, cytophosphane” obtained from CoPub.

We also compared our results with those obtained while using CoPub. Figure 3 lists CoPub results with the disease node marked in yellow, the gene node marked in gray, and the drug node marked in blue. A total of 86 genes were selected by CoPub, none of which were obtained using the proposed method.

### Scenario 2

This scenario included “acute lymphoblastic leukemia, acute myeloid leukemia” as the target disease terms and “Gefitinib, Iressa” as the target drug terms. The AML-ALL dataset reported by Golub et al. [22] was used as the disease-related GEP. This dataset contains 72 samples, each of which contains 7129 genes. We employed the drug-related GEP reported by Stegmaier et al. [23], which includes 12 samples, each containing 22283 genes.

The proposed method identified 61 dominant genes from the disease-related GEP and 191 genes from the drug-related GEP, which were then used in a search for abstracts in relation to “acute lymphoblastic leukemia, acute myeloid leukemia” in PubMed.

**Figure 4:**
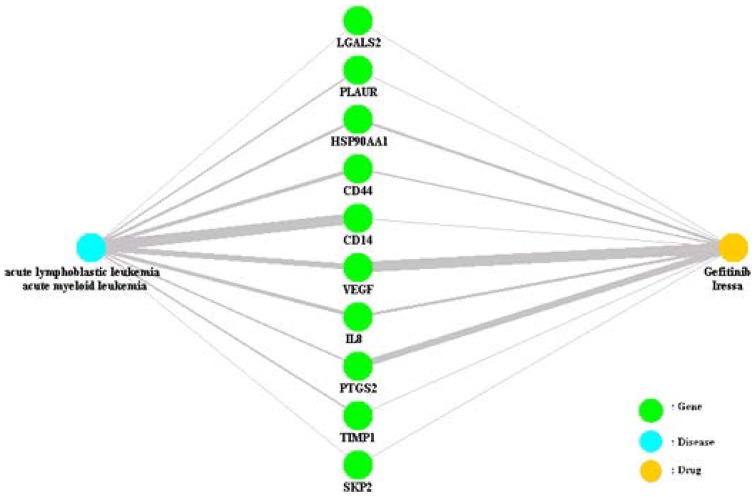
Hidden connections of the disease “acute lymphoblastic leukemia, acute myeloid leukemia” and the drug “Gefitinib, Iressa” obtained using the proposed method.

In this experiment, we identified 10 genes with hidden connections between the disease terms “acute lymphoblastic leukemia, acute myeloid leukemia” and the drug “Gefitinib, Iressa”, as shown in Fig. 4.

Gene CD14 represents an intermediate node between the disease and the drug. Yu et al. [24] claimed that “the findings suggest that CD14-260C/T polymorphism can contribute to B-ALL risk in a Chinese population”. In addition, Noh et al. stated that “Gefitinib induced the expression of differentiation markers including CD11b and CD14 in ATO-treated NB4 cells and facilitated Arsenic trioxide (ATO)-induced morphologic changes and reactive oxygen species (ROS) generation” [25]. These statements attest to the relationships between the diseases and genes as well as to that between the drugs and genes.

**Figure 5:**
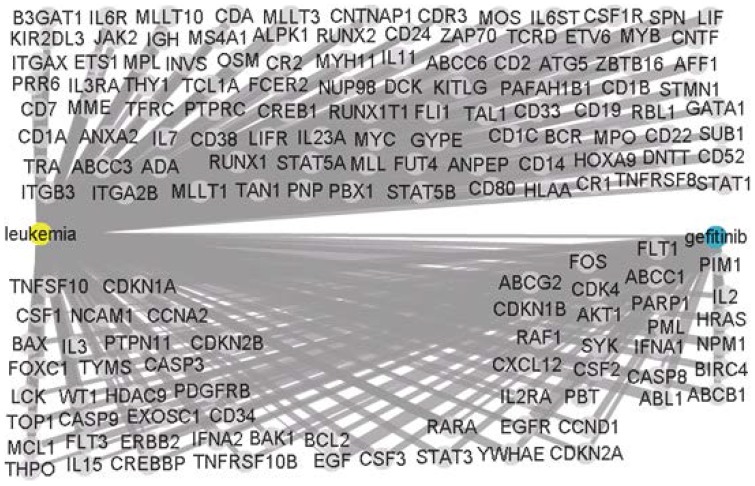
Connectivity map of the disease “acute lymphoblastic leukemia, acute myeloid leukemia” and the drug “Gefitinib, Iressa” obtained from CoPub.

Figure 5 presents the results obtained by inputting the same disease and drug terms into CoPub. It should be noted that the terms “acute lymphoblastic leukemia, acute myeloid leukemia” are classified by CoPub under the catalog term “leukemia”. As a result, the disease node in Fig. 5 is labelled “leukemia”. A total of 159 genes were selected by CoPub; however, none of these match those obtained using the proposed method.

Figure 2 (b) presents the ROC results of Scenario 2 with sensitivity and specificity of 92.0% and 83.1%, respectively. AUC in this case was 0.877 with a 95% CI of between 0.856 and 0.899.

### Scenario 3

Not every drug has GEP; therefore, this scenario simulated the situation in which only the disease-related GEP is used to obtain the list of disease-related dominant genes using DOFA. The drug-related gene list was compiled from the PharmGKB website, which provides a great deal of drug-related and gene-related information. This scenario used the terms “liver cancer”, “hepatic cancer”, and “hepatocellular carcinoma” as the target disease terms and “R340” and “capecitabine” as the target drug terms. The dataset provided by Chen et al. [26] was used as the input GEP. This dataset contains 156 samples, each comprising 3964 genes.

**Figure 6:**
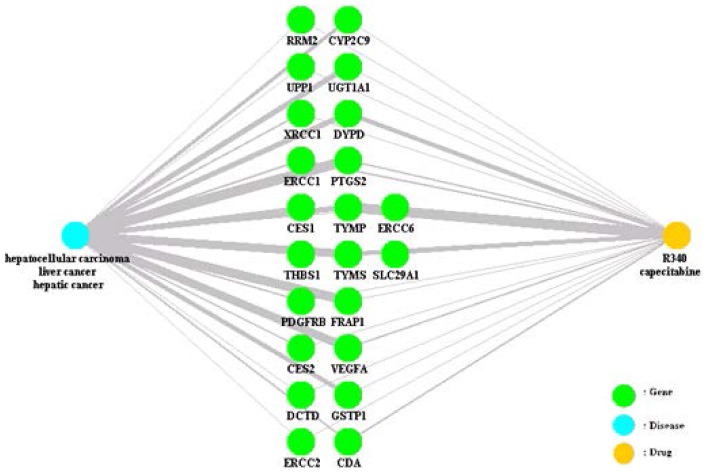
Hidden connections of the disease “liver cancer, hepatic cancer, hepatocellular carcinoma” and the drug “R340, capecitabine” obtained using the proposed method.

The proposed method identified 65 genes from the GEP and 30 genes from the PharmGKB website, which were used to identify target drug-related abstracts from the PubMed website. Figure 6 presents the 22 genes with hidden connections among the input diseases and drugs.

The gene “thymidine phosphorylase” (TYMP) represents an intermediate node between the disease and the drug. Ezaki et al. [27] claimed that “the measurement of thymidine phosphorylase (TP) activity in normal liver tissue adjacent to hepatocellular carcinoma (HCC) may predict multicentric recurrence a long time after an operation.” Ko et al. [28] also stated, “thymidine phosphorylase (TP) is the rate-limiting enzyme for the activation of capecitabine (pro-drug of fluorouracil) and a useful predictor of tumor response to capecitabine-based chemotherapy.” Clearly, the gene “TYMP” represents a hidden connection between the disease and the drug.

**Figure 7:**
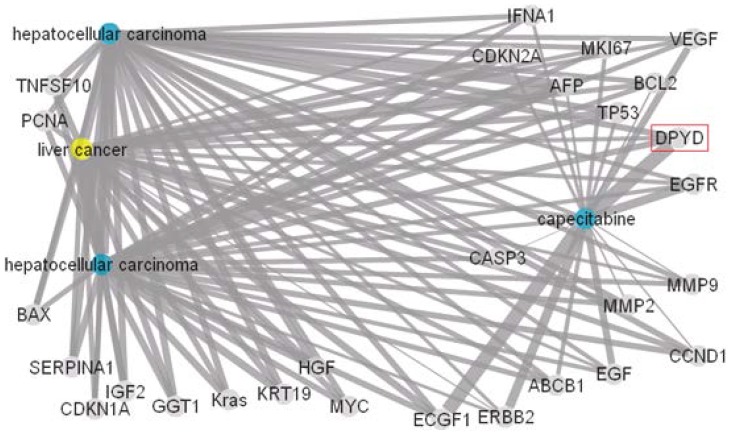
Hidden connections of the disease “liver cancer, hepatic cancer, hepatocellular carcinoma” and the drug “R340, capecitabine” obtained from CoPub.

Figure 7 presents the results of inputting the same terms into CoPub. It should be noted that the term “liver cancer” is classified by CoPub as a disease term; however, “hepatocellular carcinoma” is classified as “tissue”. Hence, in Figure 7, the disease node labelled “liver cancer” is marked in yellow, “hepatocellular carcinoma” is marked in blue, and the drug node “capecitabine” is also marked in blue. A total of 28 genes were selected by CoPub, but only one of these was obtained using the proposed method.

Figure 2 (c) shows the ROC results of Scenario 3 with sensitivity and specificity of 86.0% and 83.1%, respectively. AUC in this case was 0.833 with a 95% CI of between 0.819 and 0.848.

## Discussion

**Figure 8:**
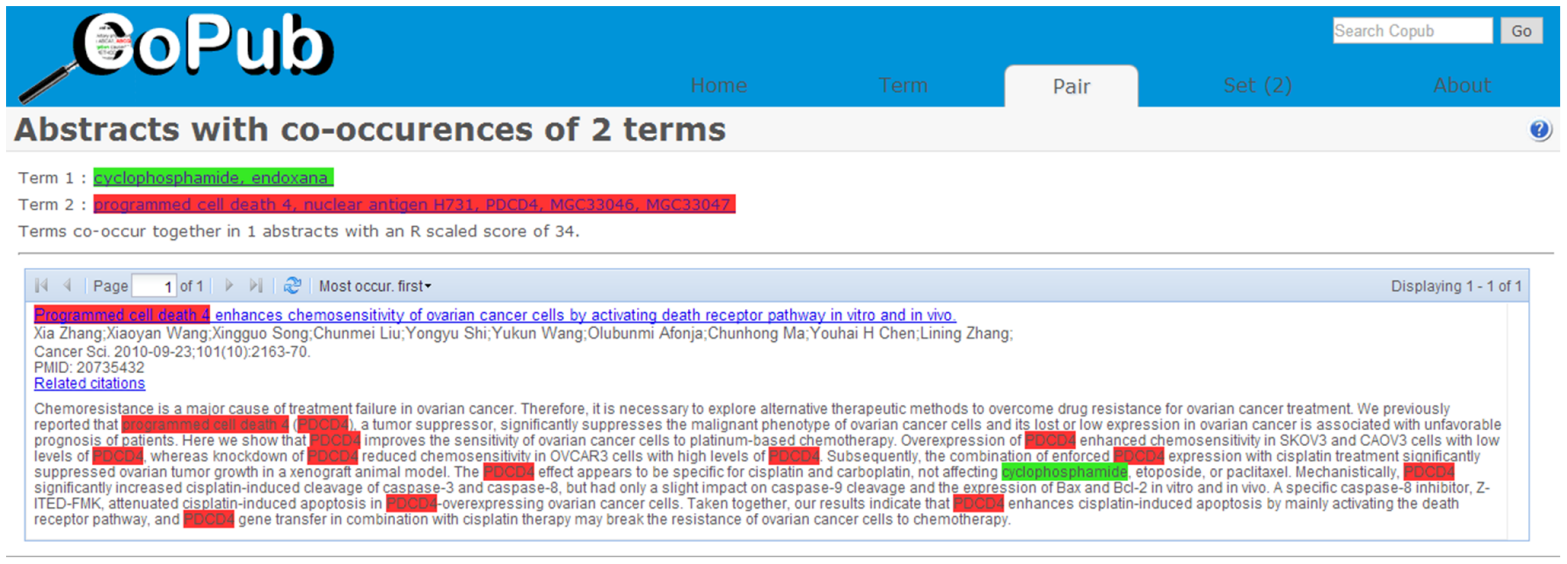
Example abstract exhibiting a negative relationship.

Uncovering hidden connections among diseases, genes, and drugs revealed that many of the retrieved abstracts were reported as negative relationships or non-relationships. For example, the co-occurrence method retrieved one study [29] pertaining to the relationship between the gene “PDCD4” and the drug “cyclophosphamide”, as shown in Figure 8. Although this study contains both of these terms, it describes a non-relationship in which the gene is not directly influenced by the drug.

The three scenarios presented a large number of documents providing negative relationships or non-relationships. Scenario 1 presented 17 hidden connections before negative-term filtering and 15 hidden connections after filtering. In Scenario 1, the proposed algorithm fetched 4028 documents (abstracts and titles). To evaluate the performance of the negative-term filtering scheme, we used the proposed weight function to verify whether these fetched documents were related to the input terms (gene or disease name), resulting in the retrieval of 834 from the 4028 documents. Then the negative-term filtering scheme removed 126 of these 834 documents. Among the removed documents, 97 were deemed to be correctly identified as “negative”, demonstrating the high performance of the negative-term filtering scheme. One example of false positives is presented by documents indicating relationships between the genes “PDCD4”and “STAT1” and the drug “cyclophosphamide”, which were subsequently removed. Another example involves “STAT1”, which recovered an abstract reporting the following: “Cs-A and IFN-gamma, but not glucocorticoids, **cyclophosphamide**, or azathioprine, inhibited TGF-beta-induced signaling, as assessed by luciferase reporter gene assays, and collagen deposition. In contrast, the effects of IFN-gamma required signal transducer and activator of transcription **(STAT)-1**” [30]. Clearly, no direct relationship is indicated between the drug “cyclophosphamide” and the gene “STAT1”. This abstract implied a non-relationship and was therefore removed.

In Scenario 2, 11 hidden connections were obtained prior to negative-term filtering. The weight function removed all but 789 documents of the 1216 documents initially retrieved and negative-term filtering removed an additional 89 documents. Among these 89 documents, 38 were deemed to be correctly identified. One example of false positives in Scenario 2 is presented by documents indicating relationships between the gene “CCNE2” and the drug “gefitinib.” These were judged to evidence a negative relationship or non-relationship, and thus the connection was removed. For example, one abstract stated the following: “combination studies revealed that the response of EGFSP genes to luteolin was not affected by **gefitinib**, even though the two compounds were additive with respect to their abilities to inhibit CCNA2, **CCNE2**, CDC25A and PCNA” [31]. Clearly, this does not indicate a direct relationship between the gene “CCNE2”and the drug “gefitinib”.

In Scenario 3, 23 hidden connections were identified prior to negative-term filtering. The weight function removed all but 1928 documents of the 3791 documents initially retrieved and negative-term filtering removed an additional 265 documents. Among these 265 documents, 202 were deemed to be correctly identified. The abstracts indicating a relationship between the input terms were judged as negative relationships or non-relationships by the negative-term filtering scheme and subsequently removed. For example, one abstract stated the following: “Taq1 digestion of PCR products revealed that both alleles were transcribed in all samples where both were presented at the genomic level, indicating that the **RRM1** locus is not subjected to imprinting in Wilms' tumour or **hepatoblastoma**” [32]. This clearly indicates that RRM1 is not subject to imprinting in hepatoblastoma, a type of liver cancer. The results of the three scenarios are summarized in Table 1.

**Table 1:** Summaries of performances of the negative-term filtering scheme of three scenarios.

	Scenario 1	Scenario 2	Scenario 3
Total # of fetched document	4028	1216	3791
# of remained documents after filtering using weight function	834	789	1928
# of related documents among the remained documents (gold standard)	500	657	1335
# of unrelated documents among the remained documents (gold standard)	334	132	593
# of true positive of remained documents judged by the negative-term filtering scheme	471	606	1272
# of false positive of remained documents judged by the negative-term filtering scheme	237	94	391
# of true negative of remained documents judged by the negative-term filtering scheme	97	38	202
# of false negative of remained documents judged by the negative-term filtering scheme	29	51	63

The first row presents the total number of retrieved documents; the second row lists the documents remaining following application of the weight function; the third and fourth rows list the number of related and unrelated documents checked by manual; and the last four rows list the number of true positives, false positives, true negatives, and false negatives, respectively. In this case, a true positive refers to a document correctly judged to be related by the negative-term filtering scheme. A false positive represents a document which is deemed to be related by the negative-term filtering scheme, but is in fact unrelated. A true negative represents a document which is correctly deemed to be unrelated by the negative-term filtering scheme. A false negative represents a document which is judged to be unrelated by the negative-term filtering scheme but is in fact related.

We compared the performance of the algorithm using the weight function and the negative-term filtering scheme. Table 1 lists only the references determined by the weight function. The negative-term filtering scheme was then performed for a second check to obtain the following results: true positive, false positive, true negative, and false negative.

Table 2 lists all retrieved references acquired using 1) the weight function and 2) using both the weight function and negative-term filtering.

**Table 2:** Comparison between only using the weight function and using both the negative-term filtering scheme and the weight function of three scenarios.

	Scenario 1	Scenario 2	Scenario 3
Total # of fetched document	4028	1216	3791
# of related documents among the fetched documents (gold standard)	584	659	1479
# of unrelated documents among the fetched documents (gold standard)	3444	557	2312
# of true positive judged by using **only the weight function**	500	657	1335
# of false positive judged by using **only the weight function**	334	132	593
# of true negative judged by using **only the weight function**	3110	425	1719
# of false negative judged by using **only the weight function**	84	2	144
# of true positive judged by **the weight function and the negative-term filtering scheme**	471	606	1272
# of false positive judged by **the weight function and the negative-term filtering scheme**	237	94	391
# of true negative judged by **the weight function and the negative-term filtering scheme**	3207	463	1921
# of false negative judged by **the weight function and the negative-term filtering scheme**	113	53	207

While performing these experiments, one problem was observed in the calculation of similarity scores among the genes and biological terms. Although synonymous gene names were obtained from GeneBank, still others were encountered due to the idiosyncrasies of individual authors' writing styles. For example, a number of authors referred to the gene “IL8” as “IL-8”, others wrote “IL 8”, and still others wrote “(IL) 8”. To compensate for this kind of variation, we developed an alias expansion scheme to accommodate a variety of styles.

For example, the alias expansion scheme enables the generation of eleven different styles for the gene “IL8”: “(IL) 8”, “IL 8”, “(IL)-8”, “IL8”, “IL-8”, “(IL) -8”, “(IL)-8”, “(IL) _8”, “IL-8”, “IL_8” and “(IL)_8”. The details of the alias expansion scheme are described in Scheme S1.

Nonetheless, there remain a number of situations that the alias expansion scheme is incapable of resolving. For example, in the sentence “we have assigned the most probable six-locus haplotypes determined by HLA-A, -Cw, -B and -DRB1 highly polymorphic genes”, we find a term “HLA-A” before the term “HLA-B”, in which both terms share “HLA”; however,“ HLA-B” is represented as “-B”. This situation can result in an erroneous classification of the relationship between the gene “HLA-B” and the disease or the drug. This complication will have to be resolved if the hit rate is to be improved.

The results obtained using the proposed method differ considerably from those obtained using CoPub. CoPub uses six thesauri containing human genes, Gene Ontology biological processes, liver pathologies, diseases, pathways, and drugs to search MEDLINE XML files containing the title, abstract and substances. CoPub stores the context and PubMed identifiers (PMIDs) of the abstracts in which the keywords are identified and then computes the strength of the hidden relationship between A and C using the R-scaled scores between A and B, and between B and C. An R-scaled score above a set threshold is regarded as biologically significant. In contrast, the proposed method uses GEPs manufactured using intermediate nodes (genes) identified by experts through experimentation.

The proposed method also handles keywords in a different manner. CoPub uses catalog terms (such as “Leukemia”) instead of the original term “acute lymphoblastic leukemia” as well as the full name of the gene, symbols, and aliases. The proposed method uses the original terms (disease or drug names) with the full name of the gene, symbols, and aliases. Although the search range of catalog terms appears to be wide, the fact is that some important information is missing. For example, CoPub uses the catalog term “colon cancer” instead of the original term “colorectal cancer” as a disease term, leading to a failure on the part of CoPub to include abstracts containing the input term “colorectal cancer”.

**Figure 9:**
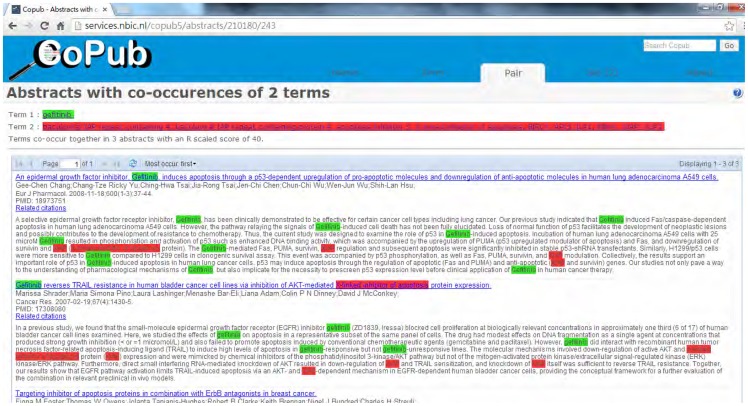
The abstracts retrieved by Copub.

In summary, these two approaches differ with regards to the source of the intermediate nodes as well as the queries. Consequently, the results also differ. Furthermore, CoPub uses only co-occurrences without considering the effect of negative relationships. For example, the abstracts [33]–[35] retrieved by Copub indicate a negative relationship or non-relationship between the gene “BIRC4” and “gefitinib” in Figure 9.

A number of the hidden connections in the connective map generated by the proposed method are thin, due to the small number of papers that mention this connection. Nonetheless, these connections may still possess biological or medical significance. In Scenario 3, the proposed method identified a hidden connection between the disease “hepatocellular carcinoma” and the drug “capecitabine”, which is an important relationship confirmed in [36].

Regardless of the method used, only a portion of the hidden connections can be identified. Both methods are based on studies performed by experts; however, if the basic relationships between A and B or between B and C are not established, neither method can provide very much in the way of helpful information regarding the hidden connections.

## Methods

**Figure 10:**
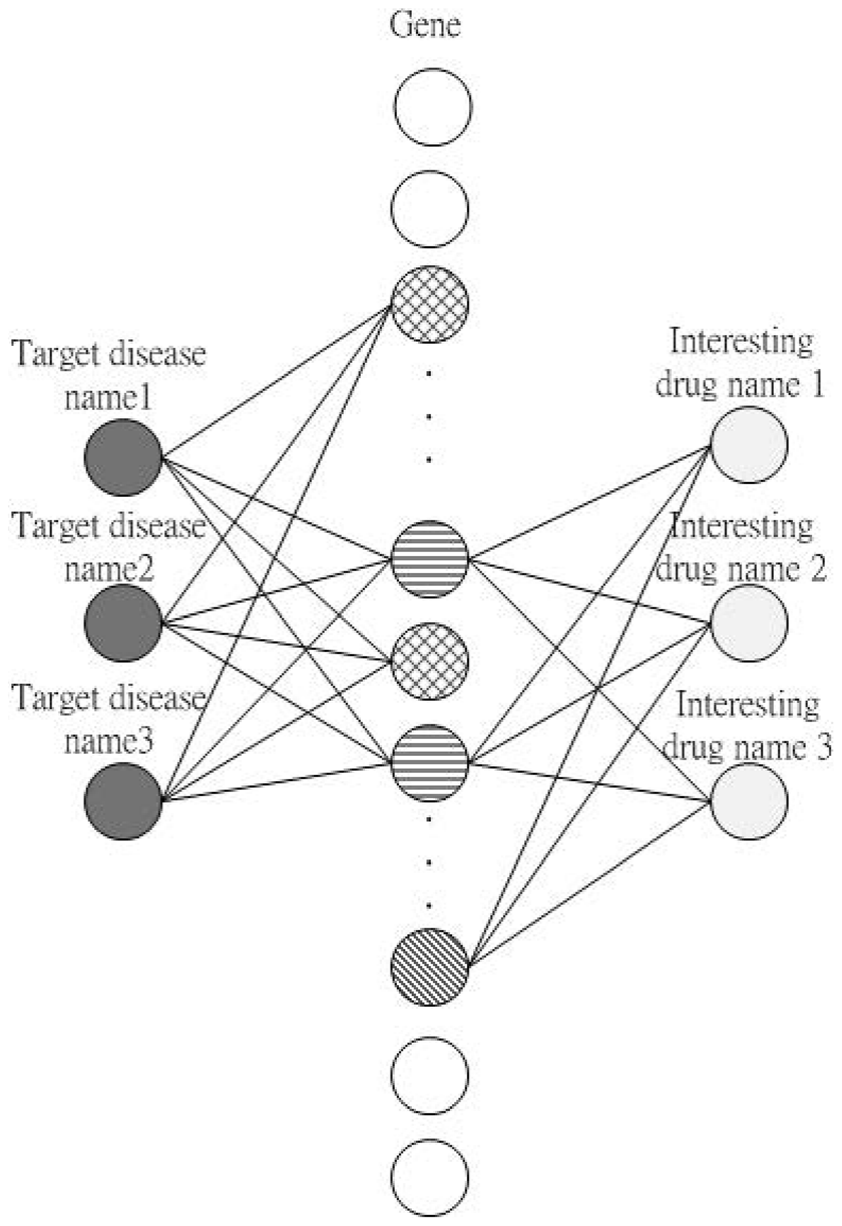
Concept behind the proposed method. Given names of target diseases and drugs and the related microarray expression profile, the proposed method searches the abstracts of genes related to the diseases and drugs, finds common parts, and provides the user a visual report.

As illustrated in Fig. 10, the objective behind the proposed methodology was the discovery of hidden connection between diseases and drugs. In Fig. 10, the left area provides target disease names such as “colon cancer” and “colorectal cancer”, and the right part lists drug names such as “5-fluorouracil” and “5-FU”. If one gene is related to a particular disease, then a line is connected from the disease to the gene. Similarly, if one gene is related to a drug, then a line is connected from the drug to the gene. Any gene related to both a disease and drug is considered a hidden connection between the disease and drug.

**Figure 11:**
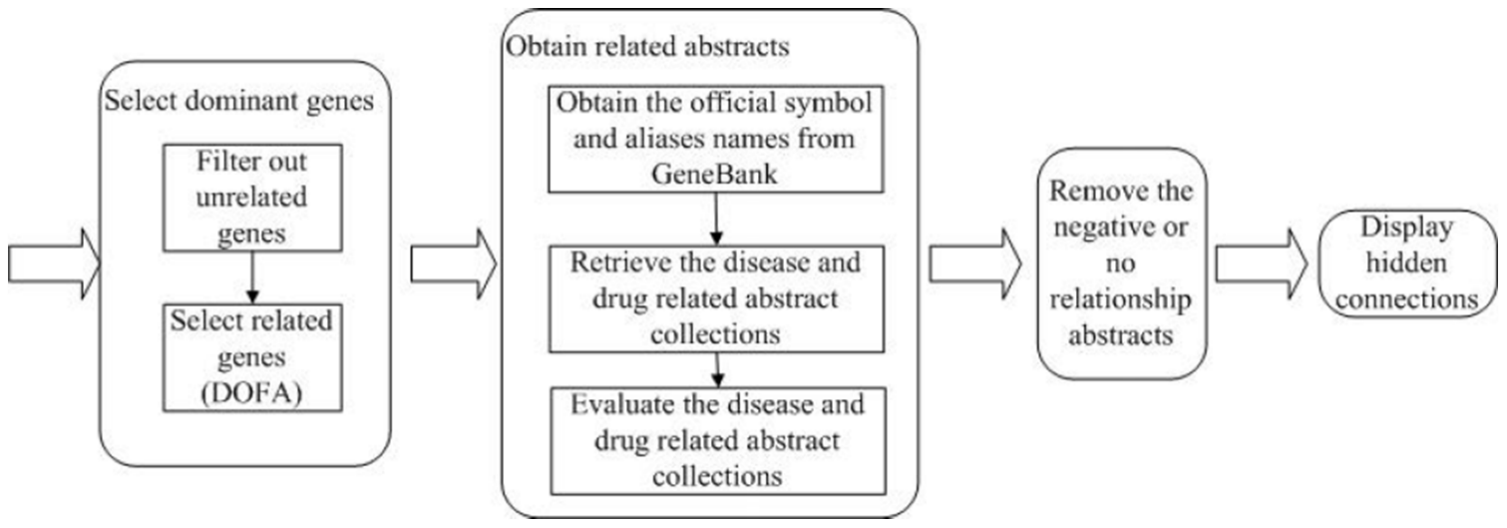
Flowchart of the proposed method.

Figure 11 presents a flowchart of the proposed method divided into four stages: feature selection, collection of related information, relationship filtering, and display of results. Feature selection (performed by the disease-oriented feature selection algorithm, abbreviated as DOFA) [18] is used for the selection of disease- or drug-related gene lists followed by a search for aliases and official symbols of the related genes from within the GeneBank database (http://www.ncbi.nlm.nih.gov/sites/entrez?db=gene). These official names and alias names are used to generate corresponding queries comprising Boolean operators with genes, diseases, and drug names. These queries are used to retrieve abstracts and titles related to these genes and the specific disease or drug names via a search on PubMed. The proposed method uses a weight function to evaluate the retrieved abstracts in order to remove those that are unrelated. Differences in the writing styles of authors can lead to discrepancies in the notation of many genes. Differences in writing style are easily overlooked if only the relationships between the retrieved abstracts and official names and aliases are considered. Therefore our proposed method includes an alias expansion scheme. In the third stage, a negative-term filtering scheme is used to verify whether the retrieved abstracts present a positive relationship, negative relationship, or non-relationship. After obtaining a collection of abstracts with gene/disease links and gene/drug links, genes occurring in two collections of abstracts are output as hidden connections. The weight function, alias expansion scheme, and negative-term filtering scheme are outlined in the following.

### The Weight Function

The abstracts retrieved in any search are not necessarily related to the disease, gene, or drugs in question. For example, when searching through documents related to “chronic myeloid leukemia (CML)”, the results may include documents related to “chronic lymphocytic leukemia (CLL)”. This makes it necessary to evaluate the abstracts collected. To calculate the relevance of the abstract, the specific disease names, and the commonplace terms (names or aliases of the specific gene) related to the specific disease, a weight function *W*(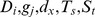) is used to calculate the related score among abstract *D_i_*, specific name *d_x_*, and gene *g_j_*, defined as follows:
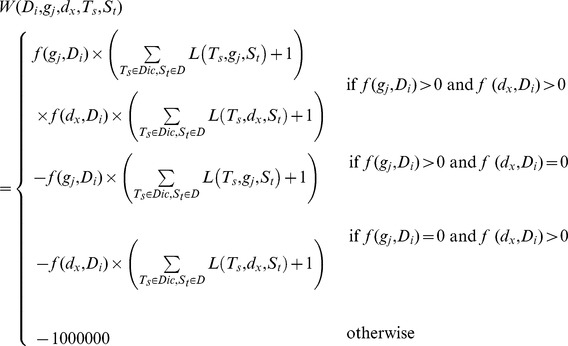
where the term frequency function *f*(*t, D_i_*) returns the number of times the variable *t* appears in abstract *D_i_*. Variable *t* can be the name of a gene or its alias, a drug name, or a disease name. Function *L*(*T_s_*, *t, S_t_*) represents the weight of the term *t* in the sentence *S_t_* and is denoted as follows:



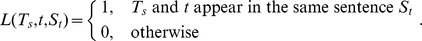



This function is used to enforce the weight of term *t* when this term and a special term *T_s_* of the special biological dictionary *Dic* appear in the same sentence *S_t_*. The special biological dictionary *Dic* is a collection of words from ten thousand cancer-related studies with the common words removed. If there is no special term *T_s_* in sentence *S_t_*, but only term t, then *L*(*T_s_*, *t, S_t_*) would return zero. If all *L*(*T_s_*, *t, S_t_*) return zero, then the weight functions would return zero without any special term. Hence, we add one to the weight function. After obtaining the related score via the weight function *W*(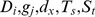), the unrelated literature is removed from the collection of abstracts. If the related score of a document calculated using the weight function is greater than zero, then this document is regarded as related; otherwise, this document is regarded as unrelated and filtered out. For example, document *D* reports the following: “Two **HLA-B** mismatches were also *significantly associated* with **lymphoma** in the kidney (HR 2.82, P = 0.009)”. Sentence *S_t_* is the same as *D*, since there is only one sentence in *D*. Gene *g* “HLA-B” appears once in *D*, hence, the *f*(*g*, *D*) returns one; for the specific name *d* “lymphoma”, *f*(*d*, *D*) gives one. Term *Ts* “significantly” and “associated” appear once in *S_t_*. Hence, the weight score of this document can be calculated as follows:
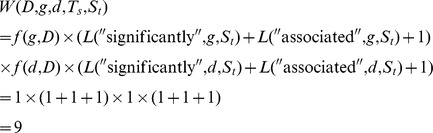



### Negative-term Filtering Scheme

The proposed algorithm seeks hidden connections within medical abstracts, yet abstracts containing negative relationships or non-relationships may result in false positives. Abstracts containing negative relationships or non-relationships should be removed to ensure the validity of the hidden connections between diseases and drugs.

**Figure 12:**
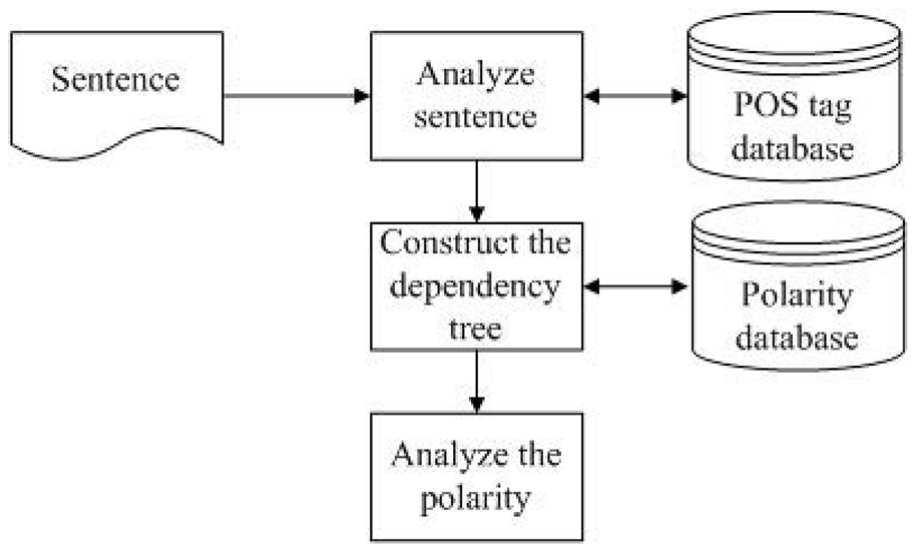
Flowchart of the negative-term filtering scheme.

To remove documents with negative relationships or non-relationships, we adopted the algorithm proposed for sentiment analysis in [37]. Figure 12 presents a flowchart of the negative-term filtering scheme. First, sentences containing input terms, such as “lymphoma cancer”, are parsed into individual words. Each of these words is then checked for the most plausible POS tag from the POS tag database and the input terms are combined into a single term. These combined terms do not necessarily appear in the POS tag database and may refer to disease terms, drug terms, or gene names; therefore, a POS tag is assigned to the input term as “NN”. A dependency tree of the sentence is then constructed in accordance with the algorithm proposed by McDonald et al. [38]. The words in each sentence are treated as vertices with the potential dependency of each word providing edges. Each edge is assigned a weight based on the possibility of two words appearing immediately after, which is used in the construction of graph G, representing the sentence. Some of the words may be found in multiple lexical categories (noun, adjective, adverbs); therefore, this graph is used to create the maximum spanning tree (MST) by maximizing number of ways that each term could be used in the sentence. This can be accomplished by determining the probability that a given term will appear in other locations, in accordance with the lexical categories to which it may belong. We then assign the corresponding polarity of each word in the resulting dependency tree via the polarity database from the leaf nodes to the root node. The policy for combining the nodes is included in Table 3.

**Table 3:** The policy for combining the nodes used in the negative-term filtering scheme.

	Parent node (Positive)	Parent node (Negative)	Parent node (Neural)
Child node (Positive)	Positive	Neutral	Neutral
Child node (Negative)	Neutral	Negative	Negative
Child node (Neural)	Positive	Negative	Neutral
Child node (Anti-)	Negative	Positive	Neutral

In this process, the term “anti-” inverts the polarity of the term to follow. For example, the two words “not elicit” imply “anti-” and “positive”. The combined polarity of these two words is therefore negative. The proposed algorithm repeats this process of combination until reaching the root node which contains a single final polarity. This final polarity represents the polarity of this sentence with regard to the input term (disease or drug name).

**Figure 13:**
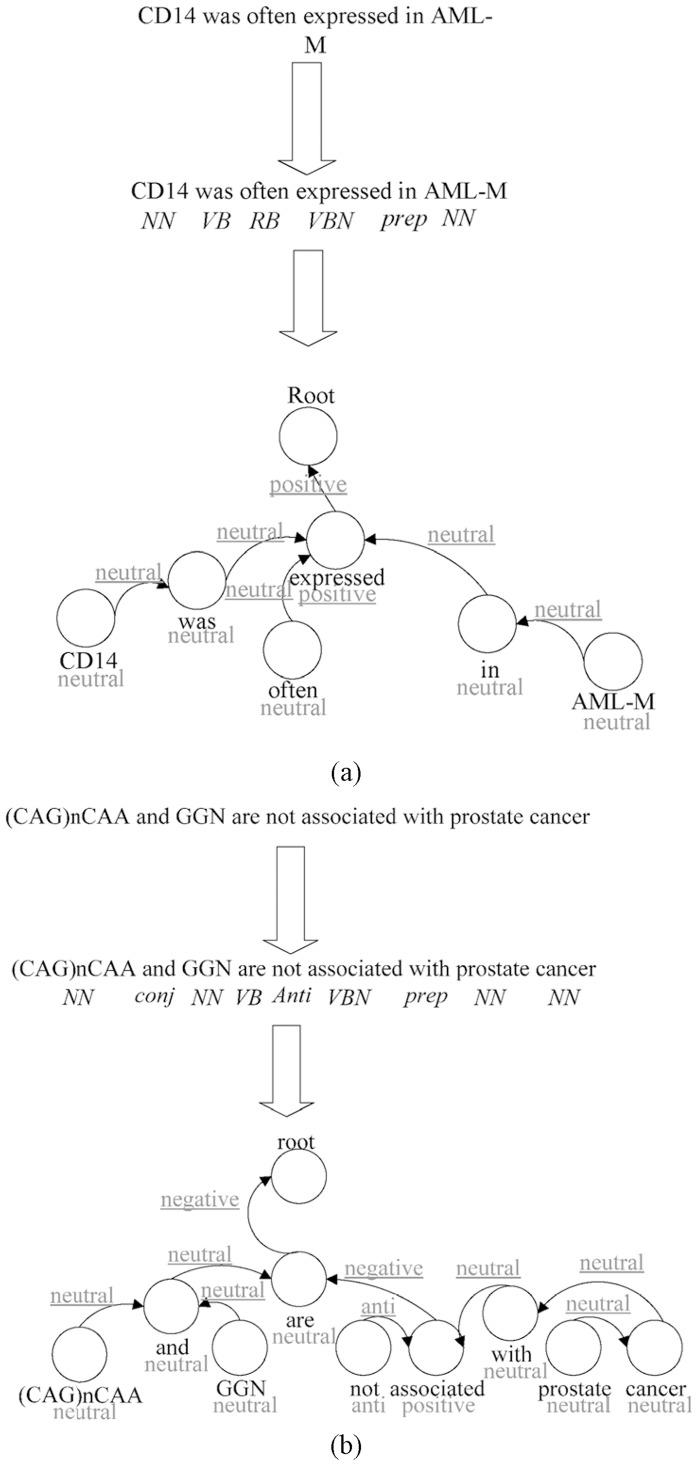
Examples of the negative-term filtering scheme.

Figure 13 presents two examples of the negative-term filtering scheme. In Fig. 13 (a), we used the sentence “CD14 was often expressed in AML-M”: CD14 and AML-M are the input terms. The POS tagging results are “NN VB RB VBN prep NN”, where NN represents a noun, VB represents a verb, RB represents an adverb, VBN represents the past participle of one verb, and prep represents preposition. The polarities of all words are then combined in accordance with the POS tagging results. In Fig. 13 (a), the polarity term is drawn in gray, and the combined polarity results are underlined. For example, the polarity of “CD14” is neutral and the polarity of “was” is neutral. When combined, these two polarity terms output a new polarity “neutral”. The polarity of “expressed” is positive and the polarities of the other three combined results are all neutral, such that the root node outputs a final combined result of “positive”. Figure 13(b) presents an example of negative polarity derived from the sentence “(CAG)nCAA and GGN are not associated with prostate cancer”.

It should be noted that the two databases used in the negative-term filtering scheme were constructed in advance. The POS tag database was constructed using the renowned Brown corpus [39]. The polarity database was manually constructed by defining the polarities of all words in the POS tagging database. The meaning of each word was looked up in the dictionary and a group of experts was assembled to assign polarity to each word according to its meaning. For example, the word “better”, is listed as the comparative of “well” in the dictionary. Thus, its polarity was deemed “positive”. Any word without a clear polarity was marked “neutral”.

(The database can be downloaded in [40]).

The Brown corpus does not contain the terms “downregulate (VB)”, “downregulated (VBD)”, “downregulated (VBN)”, “upregulated (VBN)”, “upregulated (VBD)”, and “upregulate (VB)”, yet these terms are important in the extraction of relationships from documents; therefore, we manually inserted these terms into the polarity database. These six words also follow the same rule to assign their polarities of words. In addition, terms that were not found in the polarity database were automatically assigned a “neutral” polarity.

Although the proposed negative-term filtering scheme is able to filter out a large number of documents with negative relationships or non-relationships, there remains considerable room for improvement through the inclusion of additional negative-terms and semantic analysis of greater subtlety.

### Alias Expansion Scheme

**Figure 14:**
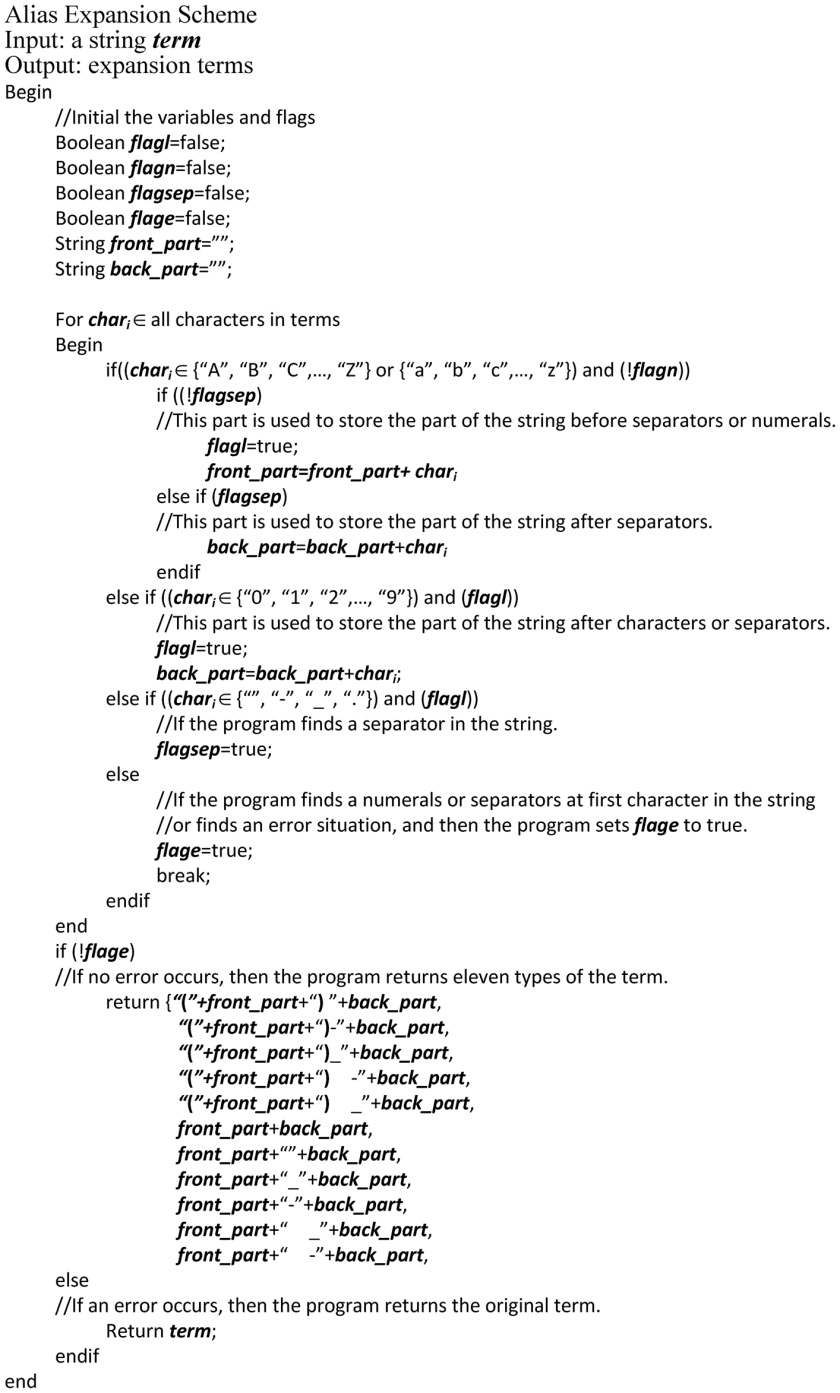
Pseudo-code of the alias expansion scheme.

The weight function uses the frequency with which the names of input genes appear in the retrieved abstracts in order to evaluate the relevance of the documents. This necessitated developing the means to generate a comprehensive list of gene formats to accommodate variations in the writing styles of authors and subsequently minimize the possibility of misjudging the weights in the proposed algorithm. Figure 14 shows the pseudo code of the alias expansion scheme, which is presented in further detail in Scheme S1.

## Supporting Information

Figure S1 **The flowchart of DOFA.**
(TIFF)

Figure S2 **Some of original data of the Colon dataset.**
(TIFF)

Figure S3 **Flowchart of the classification pattern learning phase of the proposed algorithm.**
(TIFF)

Figure S4 **An example of the classification pattern** ***ID*** **for class** ***k*** **.**
(TIF)

Algorithm S1 **Disease orient feature selection algorithm (DOFA).**
(DOCX)

Data S1 **Experimental Results.**
(RAR)

Scheme S1 **Alias Expansion Scheme.**
(DOCX)
