# Supplementary material for: Discovering Hidden Connections among Diseases, Genes and Drugs Based on Microarray Expression Profiles with Negative-Term Filtering
Source: PLoS One. 2014 Jun 10;9(6):e98826. doi: 10.1371/journal.pone.0098826 (PMC4051596; doi:10.1371/journal.pone.0098826)
Supplement: Algorithm S1 — Disease orient feature selection algorithm (DOFA). (DOCX) [file pone.0098826.s005.docx]

## Disease-Oriented Feature Selection Algorithm

This supplementary introduces a genetic approach for picking up related genes corresponding to the diseases. The optimized solution which refers to the best combination gene set to classify the data in a set of microarray data. However, the amount of genes in a microarray data is so huge that these genes can't be used directly as the individuals of Genetic Algorithms (GAs). Therefore, first, we check these genes to reduce the unrelated genes. If all expression values of the samples at one gene are close to the means value of their classes and far away from the other classes, then this gene is regarded as important gene. If the expression values of the samples at one gene are hard to distinguish the classes, then this gene is unimportant. After removing the unrelated genes, we adopt GA to pick up meaningful genes to the diseases as classification patterns. At the end of the algorithm, the classification patterns are used to classify the testing datasets.

## Overview of DOFA

Before describing the whole algorithm, some variables are defined first. Suppose that a training dataset *S*

$S=\{X^{j}|X^{j}=(x_{1}^{j},x_{2}^{j},...,x_{m}^{j}),1\leq j\leq n\}$ (1)

consists of *n* expression samples, and m is the number of genes measured. In *S*, all samples associated with a class label *k*∈{1,2,…,*c*} can constitute a subset *S_k_*, and

$S=S_{1}\cup S_{2}\cup...\cup S_{k}\cup...\cup S_{c}$ (2)

where *c* is the number of total classes.

DOFA consists of four processes: the normalization process, the selection process, the classification process and the fusion and verification process. The flowchart of DOFA is given in Figure S1.

First, the microarray expression training dataset *S* is normalized to eliminate the difference between scales of different genes. And then, the normalized gene expression data is sent to the gene selection process to select informative genes to the diseases. The gene selection process consists of two phases, the gene reduction and the classification pattern learning phase. In gene reduction phase, we normalize the gene expression data in order to eliminate the difference caused by the scale of gene expression. And then, we analyse the genes to filter out the unimportant genes. In the classification pattern learning phase, DOFA uses GA to further learn the classification patterns for each disease and preserves the informative genes of the training and testing datasets using the classification patterns and then sends them to the SVM to obtain the classification results. In the final process, the classification results are fused with one predicted result using a decision function to decide the testing sample to which class it belongs. The predicted results are verified to calculate the classification accuracy.

## Normalization process

Since the expression values of genes have notable differences between the scales, in order to eliminate these differences, the gene expression data should be normalized first. Note that the “normalization” here is different from the normalization process for adjusting the signals from the microarray to obtain the expression values. The “normalization process” here is to eliminate the scale differences between different genes. Figure S2 gives an example to show the real values of one microarray expression dataset.

As shown in Figure S2, the differences between the scales of different genes are large. The scale of the gene expression values of gene *x_1_* is as large as thousands, and the scale of the gene expression values of gene *x_3_* is small to tens. If we classify the microarray expression data using these un-normalized data, some genes related to disease may be neglected. Hence, a normalization process to eliminate these differences of genes is needed.

The normalized function Nor($x_{i}^{j}$) is defined as follows :

$Nor(x_{i}^{j})=\frac{x_{i}^{j}-x_{i,\min}}{x_{i,\max}-x_{i,\min}},\forall j=1,2,...,n\text{ },\text{ }i=1,2,...,m$ (3)

where *x_i,max_* represents the maximum value of all gene expression samples at *i*-th gene; *x_i,min_* denotes the minimum value of all gene expression samples at *i*-th gene. And these two values, *x_i,max_* and *x_i,min_*, are denoted as follows :

(4)

(5)

According to the normalized function, all gene expression values of training dataset are normalized to 0 ~ 1. All expression values are normalized according to the normalized function, the dataset *S* can be transformed to the normalized dataset *S’*. After normalization, the relationships between genes and classes are more easily and clearly to find. However, not all genes are the same important to all classes. Some genes are unrelated to disease and regarded as noise. Hence, the unrelated genes should be removed in order to increase the classification accuracy.

## Gene selection process

In this supplementary, the gene selection process, which is the most important core of DOFA, consists two phases, the gene reduction phase and the classification pattern learning phase. The gene reduction phase is used for filtering out the unrelated genes. The classification pattern learning phase further selects the informative genes of the disease.

## Gene reduction phase

In the gene reduction phase, it consists of 2 procedures: gene ranking procedure and gene reduction procedure. In the gene ranking procedure, the normalized training dataset *S’* is used to calculate the scores for each gene to each class using a gene ranking function. The matrix *R* records all scores of every gene to classes which are calculated using the gene ranking function. For each gene *x_i_*, $\mu_{i}^{k}$ is the mean of *i*-th genes for class *k*, it is calculated as follows:

$\mu_{i}^{k}=\frac{\sum_{\forall j,X^{j}\in S_{k}} x_{i}^{j}}{|S_{k}|}$ (6)

where |*S_k_*| represents the number of samples in the *k*-th class subset *S_k_*.

And *μ^k^* is the mean vector of all genes for the class k. *μ^k^* is denoted as follows:

$\mu^{k}=(\mu_{1}^{k},\mu_{2}^{k},...,\mu_{m}^{k})$ (7)

When the mean vector *μ^k^* is determined, a gene ranking function, *score*(*i*, *k*), indicates the ability of the *i*-th gene to identify the samples associated with the *k* class label. The gene ranking function, *score*(*i*, *k*), is defined as follows:

$score(i,k)=\frac{\left[\sum_{\forall j,X^{j}\in S_{k}} v(x_{i}^{j},k)\right]}{|S_{k}|}$ (8)

The weighted voting function *v*($x_{i}^{j}$, *k*) is given as follows

(9)

The weighted voting function measures the distance between the center of the class *k* and the expression values of *i*-th gene and the distance between the center of the class *l* (*l*∈{1,2,…,c},*l*≠*k*) and the expression values of *i*-th gene. If the expression value is close to the center of the class *k*, in other words, the distance between the expression value and the center of the class *k* is smaller than the distances between the expression value and the center of any other class, then the weighted voting function returns 1; otherwise, the function returns 0.

All scores among all genes to all classes are recorded in the matrix *R*. The higher a score is, the more ability the corresponding *i*-th gene can distinguish the samples of class *k*.

In the gene reduction procedure, the gene selection vector *T^k^*, 1≤*k*≤c which records the information of the genes whether are noise or not for class *k* is generated according to the threshold *thr* and the matrix *R*. All the gene selection vectors are recorded in the matrix *T*. For different classes, one gene may have different scores. Hence, in order to find out the genes that are related to the class k, the vector *T^k^*=($t_{1}^{k}$,$t_{2}^{k}$,…,$t_{i}^{k}$,…, $t_{m}^{k}$) is a gene selection vector that records the selected genes of class k. The attribute $t_{1}^{k}$ of the vector *T^k^* is defined as follows

$t_{i}^{k}=\left\{\begin{matrix} 1, & \text{if }score(i,k)\geq thr \\ 0, & \text{otherwise} \end{matrix}\right.$ (10)

Those genes whose scores exceed a threshold value *thr* are regarded as the related genes and preserved to the following classification pattern learning phase. On the other hand, those genes whose scores do not exceed the threshold value *thr* are regarded as the unrelated genes and removed. The threshold *thr* is used to remove the unrelated genes to narrow down the search space of the following classification pattern learning phase. Changing the value of *thr* only affects the size of the search space, and it doesn’t affect the classification accuracy. Hence, the threshold *thr* is determined by an empirical rule. Then, the normalized training dataset *S’* uses the *T* matrix to remove the unrelated genes and transforms into the attribute reduced training datasets of each disease.

## Classification pattern learning phase

Since the genetic algorithms [1] (abbreviated GAs) has good performance in clustering [2], in this supplementary, we use GAs in the classification pattern learning phase of the gene selection process to find out those meaningful classification patterns for each class. Figure S3 shows the flowchart of the classification patterns learning phase that consists of six procedures: the classification pattern generation procedure, the evaluation procedure, the selection procedure, the crossover procedure, the mutation procedure and survival procedure.

The classification patterns are generated in the first procedure. For class *k*, the classification pattern *ID*, which is the individual of GA, is a binary string whose length is $\alpha_{k}$. The value $\alpha_{k}$ is the number of genes in $S_{r}^{k}$ that is the attribute reduced training dataset according to the gene selection class *k* vector *T^k^*.

Figure S4 shows the framework of *ID*. The elements of *ID* represent the genes whether they are selected or not. When the value is “1”, it means that the corresponding gene is selected; otherwise, the gene is abandoned.

In the evaluation procedure, these classification patterns are evaluated via the fitness function. In this procedure, the *k* nearest neighbour [3] (abbreviated KNN) is used for evaluation. We proposed a fitness function that combines the KNN fitness function and the expected value fitness function to be defined in the following to evaluate the performance of the classification pattern *ID*.

$fitness\_c(ID,k,S_{r}^{k'})=fitness\_e(ID,k,S_{r}^{k'})\times fitness\_k(ID,S_{r}^{k'})$ (11)

According to the content of *ID*, the evaluation phase selects the genes of the attribute reduced training dataset $S_{r}^{k}$ to form the reduced dataset $S_{r}^{k'}$. The fitness function $fitness\_e(ID,k,S_{r}^{k'})$ indicates the ability of the *ID* to identify the samples associated with the *k* class label, and it is defined as follows:

$fitness\_e(ID,k,S_{r}^{k'})=prob(ID,k)\times CR(k,S_{r}^{k})+(1-prob(ID,k))\times CE(k,S_{r}^{k'})$ (12)

The function *prob*(*ID*, *k*) returns the probability of accuracy with each sample of the subset $S_{rk}^{k'}$ of $S_{r}^{k'}$ in class *k*, and it is defined as follows:

$prob(ID,k)=\frac{\left(\sum_{\forall z,\overrightarrow{P_{z}}\in S_{rk}^{k'}} g(\overrightarrow{P_{z}},k)\right)}{|S_{rk}^{k'}|}$ (13)

and $g(\overrightarrow{P_{z}},k)$ is defined as follows:

$g(\overrightarrow{P_{z}},k)=\left\{\begin{matrix} 1, & \text{if }\left\|\overrightarrow{P_{z}}-\overrightarrow{\mu^{k}}\right\|<\left\|\overrightarrow{P_{z}}-\overrightarrow{\mu^{l}}\right\|,\forall l\in\{1,2,...,c\},l\neq k \\ 0, & \text{otherwise} \end{matrix}\right.$ (14)

where $\overrightarrow{P_{z}}$ is the sample that belongs to subset $S_{rk}^{k'}$. The center vector $\overrightarrow{\mu^{y}}$ denoted in the following is the average value of all genes for the class *y* in the reduced dataset $S_{r}^{k'}$.

$\overrightarrow{\mu^{y}}=(\mu_{1}^{y},\mu_{2}^{y},...,\mu_{\beta_{k}}^{y}),1\leq y\leq c$ (15)

where $\beta_{k}$ is the number of genes in reduced dataset $S_{r}^{k'}$. The values of *CR*(*k*, $S_{r}^{k'}$) and *CE*(*k*, $S_{r}^{k'}$) are denoted as

$CR(k,S_{r}^{k'})=\left(\sum_{\forall z,\overrightarrow{P_{z}}\in S_{rk}^{k'}} g(\overrightarrow{P_{z}},k)\right)$ (16)

$CE(k,S_{r}^{k'})=|S_{rk}^{k'}|-\left(\sum_{\forall z,\overrightarrow{P_{z}}\in S_{rk}^{k'}} g(\overrightarrow{P_{z}},k)\right)$ (17)

The KNN fitness function, *fitness_k*(*ID*, $S_{r}^{k'}$)$fitness\_k\left( ID, S_{r}^{k'} \right)$, is denoted as follows:

$fitness\_k(ID,S_{r}^{k'})=KNN(ID,S_{r}^{k'})$ (18)

The $KNN(ID,S_{r}^{k'})$ function returns the accuracy that is obtained by using the KNN to classify the reduced dataset $S_{r}^{k'}$.

When obtaining the fitness values of all classification patterns, the maximum fitness value *fitness_max_*$\mathrm{fintess}_{\max}$ and the average fitness value *fitness_avg_* $\mathrm{fitness}_{\mathrm{avg}}$are found and used in calculating the crossover rate and the mutation rate.

In the selection procedure, the competing method is adopted to select classification patterns for the following two procedures, the crossover procedure and the mutation procedure to generate new offsprings.

In the crossover procedure, each pair of parents performs crossover with the crossover rate in the paper [4] is denoted as follows:

$\text{crossover rate}=\frac{fitness_{\max}-fitness'}{fitness_{\max}-fitness_{avg}}$ (19)

where the fitness’ is the maximum fitness value of the selected two individuals, *A* and *B*, and is defined as follows:

$fitness'=\max(fitness_{A},fitness_{B})$ (20)

In this paper, the one point crossover method is adopted in the crossover phase.

In the mutation procedure, each classification pattern of offspring mutates with a probability. The mutation rate in the paper [4] is adopted in the mutation phase and is denoted as follows:

$\text{mutation rate}=\frac{fitness_{\max}-fitness_{i}}{fitness_{\max}-fitness_{avg}}$ (21)

where *fitness_i_* is the fitness value of *i*-th classification pattern of offspring. In this paper, the two points mutation is adopted. The two elements of one classification pattern are randomly selected, and the values of the elements are inversed, i.e. if the value is “1”, it changes to “1”, vice versa.

When collecting enough offsprings, these new classification patterns of the offsprings are further evaluated. And then, the global best is updated according to these fitness values of all classification patterns. In the final procedure, the enhanced roulette wheel method is used to preserve the population into next generation. The classification patterns are sorted and ranked according to the fitness value. And then, [the](http://tw.dictionary.yahoo.com/search?ei=UTF-8&p=%E5%AD%98%E6%B4%BB%E7%8E%87) [survival](http://tw.dictionary.yahoo.com/search?ei=UTF-8&p=%E5%AD%98%E6%B4%BB%E7%8E%87) [rate](http://tw.dictionary.yahoo.com/search?ei=UTF-8&p=%E5%AD%98%E6%B4%BB%E7%8E%87) of each classification pattern is calculated. If the fitness value is higher, the classification pattern has higher probability to survive the next generation.

## Classification process

When selecting informative genes is finished, the training dataset and the testing dataset use the matrix T to remove the unrelated genes. And then, the reduced training and testing datasets use the classification patterns to select the informative genes, and then perform the classification process. SVM uses the training dataset to learn hyperplane that is used to classify the testing dataset.

## Fusion and verification process

The proposed algorithm adopts a decision rule for fusing all the classification results and decides the testing samples to which class they belong. If all the results show that the testing sample belongs to the class A, the decision rule classifies the testing sample to class A. If no class result claims that the testing sample belongs to its class, or more than one class results claim that the testing sample belongs to their classes, then the testing sample is sent to KNN to decide which class the testing sample belongs to.

## Reference

1. J. H. Holland, *Adaptation in natural and artificial system*. University of Michigan Press (1975).
2. Y. Liu, X. R. Pu, Y. D. Shen, Z. Yi and X. F. Liao, Clustering using an improved hybrid genetic algorithm. *International Journal on Artificial Intelligence Tools* **16** (2007) pp. 919-934 DOI: 10.1142/S021821300700362X.
3. J. H. Friedman, Flexible metric nearest neighbor classification. In *Technical report Dept. of Statistics* (Stanford University 1994).
4. Y. K. Kwok and I. Ahmad, Efficient scheduling of arbitrary task graphs to multiprocessors using a parallel genetic algorithm. *Parallel and Distributed Computing* 47 (1997) pp. 58-77.

## Support Information Legends

**Figure S1 - The flowchart of DOFA**

**Figure S2 - Some of original data of the Colon dataset**

**Figure S3 - Flowchart of the classification pattern learning phase of the proposed algorithm**

**Figure S4 - An example of the classification pattern *ID* for class *k***
