# Supplementary material for: Discovering Hidden Connections among Diseases, Genes and Drugs Based on Microarray Expression Profiles with Negative-Term Filtering
Source: PLoS One. 2014 Jun 10;9(6):e98826. doi: 10.1371/journal.pone.0098826 (PMC4051596; doi:10.1371/journal.pone.0098826)
Supplement: Scheme S1 — Alias Expansion Scheme. (DOCX) [file pone.0098826.s007.docx]

## Alias Expansion Scheme

Since we use the weighted function based on co-occurrence to evaluate the relevance of the fetched back document, the times of a gene name appear in the abstract is important to our method. However, the names and aliases of some genes used in the literature may not be written in the conventional manner, for example, "IL8" can be written as "IL-8", "IL 8", "(IL) 8". When evaluating the relevance of the fetched back abstracts, these different writing styles may be missed if we only calculate the appearance of the official name and aliases of the related gene. Hence, we proposed the alias expansion scheme to solve this problem.

We used three terms “HLA-A”, “IL8”, and “CD40LG” to explain the scheme. For “HLA-A”, the scheme first searches for characters that are not letters. In this example, the character is ‘-‘. The scheme then stores “HLA” into variable “***front_part***”. The scheme ignores the non-letter character, because the first non-letter character is ‘-‘, and begins searching through the remainder of the substring for other non-letter characters. In this example, the scheme finds nothing. Hence, “A” is stored into variable “***back_part***”. Finally, the scheme returns eleven alias expansion words “(HLA) A”, “HLA A”, “(HLA)-A”, “(HLA) _A”, “(HLA) -A”, “HLA-A” , “HLAA”, “HLA_A” , “HLA-A”, “HLA_A” and “(HLA)_A”.

For “IL8”, the algorithm first searches for characters that are not letters. In this example, the character is “8“. Thus, the algorithm stores “IL” into the variable “***front_part***”. The scheme stores this non-letter character into the variable “***back_part***”, because this non-letter character is ‘8‘,and begins searching through the remainder of the substring for letters. In this example, the scheme finds nothing. Hence, the scheme returns eleven words “(IL) 8”, “IL 8”, “IL8”,“(IL)-8”, “(IL) -8”, “(IL) _8”, “IL-8”, “IL_8” , “IL-8”, “IL_8” and “(IL)_8”.

For “CD40LG”, the scheme first searches for characters that are not letters. In this example, the character is “4“. The scheme then stores “CD” into the variable “***front_part***”. The scheme stores the non-letter character “4” into the variable “***back_part***”, because this non-letter character is “4”, and searches the remainder of the substring for a letter. If a letter is found, then this gene cannot generate any expansion terms via the proposed scheme and only the original name is returned. In this example, the scheme finds ‘L’ of the gene name “CD40LG”. Hence, the scheme returns only the original name “CD40LG”.

Nonetheless, many of the expanded aliases do not have any meaning, which may cause many false-positive hits. For the IL8 gene, we obtained eleven words “(IL) 8”, “IL 8”, “(IL)-8”, “IL-8”,”IL8”, “IL_8”,“(IL) -8”, “(IL) _8”, “IL-8”, “IL_8” and “(IL)_8” via the scheme. However, the term “(IL) 8”, “IL 8”, “(IL) -8” and “(IL) _8” may retrieve studies with the term “IL”, such as “IL6”. Studies that are not related to the gene of concern are regarded as false-positive hits. Hence, the proposed method does not use the expanded aliases to fetch related abstracts, but uses these expanded aliases only to calculate similarity.
